# AI, big data, and robots for the evolution of biotechnology

**DOI:** 10.5808/GI.2019.17.4.e44

**Published:** 2019-11-13

**Authors:** Haseong Kim

**Affiliations:** 1 Synthetic Biology and Bioengineering Research Center, Korea Research Institute of Bioscience and Biotechnology, Daejeon 34141, Korea; 2 Biosystems and Bioengineering Program, University of Science and Technology, Daejeon 34113, Korea

**Keywords:** artificial intelligence, big data, ubiquitous robotic companions

## Abstract

Artificial intelligence (AI), big data, and ubiquitous robotic companions —the three most notable technologies of the 4th Industrial Revolution—are receiving renewed attention each day. Technologies that can be experienced in daily life, such as autonomous navigation, real-time translators, and voice recognition services, are already being commercialized in the field of information technology. In the biosciences field in Korea, such technologies have become known to the local public with the introduction of the AI doctor Watson in large number of hospitals. Additionally, AlphaFold, a technology resembling the AI AlphaGo for the game Go, has surpassed the limit on protein folding predictions—the most challenging problems in the field of protein biology. This report discusses the significance of AI technology and big data on the bioscience field. The introduction of automated robots in this field is not just only for the purpose of convenience but a prerequisite for the real sense of AI and the consequent accumulation of basic scientific knowledge.

Historically, artificial intelligence (AI), which is represented by deep learning, is closely related to biology. To be more precise, the relationship is between statistics and genetics. The prominent statisticians Karl Pearson, William S. Gosset, and Ronald A. Fisher, who created the most well-known statistical theories, such as correlation coefficient, *T*-test, and probability distribution, were working as a biology professor, a biologist at a brewery, and a biologist at an agricultural test field, respectively. Notably, the famous pea breeding experiment of Gregor Johann Mendel provided crucial data for the development of the statistical theories by Pearson and Fischer. Mendel discovered the Laws of Heredity, by which the genetic factors of pea plants are passed on to the offspring according to a certain set of rules to determine the offspring’s trait. All the studies by Mendel, Pearson, and Fischer are equivalent to statistical models that describe the relationship between the genetic factors and the observed traits. The term “error” in statistical models enables explaining the relationship between the two factors with a simple equation, even when their complex mechanical relationship is unknown. In fact, to build an exact mechanistic model of the hereditary DNA and its effect on the generation of yellow peas was not possible based on the existing knowledge. Every cell holds over millions of proteins and an even larger number of metabolites. Interactions between these molecules determine a trait, such as that in yellow peas. The statistical models studied by Pearson and Fischer involved only simple arithmetic operations among the observed trait probabilities; however, their genetic theories were adequately proven and accepted as general law of hereditary. Thus, a good statistical model focuses on having the least errors rather than explaining the exact mechanical relations of the observed phenomenon, and the ‘error’ in a statistical model is essential for the unknown and complex biological phenomenon.

The fundamental principle of the statistical models applies equally to AI (deep-learning) models. Initiated by Marvin Lee Minsky at the Dartmouth Conference, 1965, AI began to attract significant attention [1]. Since then, AI has undergone a steady progress in its development, with alternating periods of dark ages and revival. Nonetheless, ever since the presentation of applied cases in 2016, AI has become a representative keyword of the present era facing the 4th Industrial Revolution. There are two main reasons why the AI theory, developed before the 1960s, has gained attention recently. One is the improvement in computing performance with software and hardware related to parallel computation using GPUs or multi CPUs. The other is big data on image, text, or voice as a result of internet and personal device development. At present, the fields in which high-level deep-learning technology is available are limited to those that utilize image, video, voice, and text data. For example, the Open Image Dataset [2] provided by Google comprises approximately 15 million images and 600 categories (labels). The WMT14 dataset [3] that used to be applied in Google Translator consisted of 6 million sentences and 340 million words. High performance AI is limited to these types of datasets, because they are relatively easy to collect and label. For instance, in 2011 the accuracy of facial image recognition was approximately 75%, which was inferior compared with 97.53% accuracy of the human ability to recognize faces. However, approximately three years later, an accuracy of 97.35% was achieved by Facebook. In 2011, Facebook launched a tag suggestion service that looked for friends in photo images, whereby facial data were collected. With this service, one could simply click on a tag suggested on the face of a friend in a photo to get a set of photo data carrying a label of the friend’s name. For AI learning, such labeled data are essential, and Facebook used their web service visiting approximately 1.5 billion users per day to collect labeled large-scale facial photo data.

Compared with the image or text datasets, biological data are much more complex and multidimensional, with a high level of noise. Thus, an AI model is more appropriate than a simple statistical model for biological datasets. Generally, the main objective of biological research is to uncover the genetic factors that affect a specific trait. In terms of AI model, phenotype labeled genotype data are required but it is never easy to collect more than hundreds of thousands of labeled genotype data like the case of images or texts. A relatively easier way to collect labeled genotype data is to sequence the DNA of a sample with a definite phenotype, such as a disease. The recent advancement of next-generation sequencing (NGS) technology has allowed the systematic collection of labeled data based on phenotype. The most recent example is the EU project that is collecting genomic data of a million patients with cancer, infectious or rare diseases [4]. Fortunately, in the healthcare sector, large-scale investments, predicated on a mutual agreement on the importance of biological data, are underway. However, of the approximately one trillion or more species of other biological organisms (especially microorganisms) that have been predicted to inhabit the Earth, 99% are yet to be identified. Although NGS technology would allow rapid genomic sequencing, the processes of annotating the decoded sequences require high cost investments and increased specialist involvement. Without the improvement of the time-consuming and specialist-dependent processes of experiments for the function determination and categorization of DNA sequences, the current level of AI models would come to a standstill.

Such problems were recognized by groups researching advanced synthetic biology in the United States and Europe, who went on to apply automated robots to carry out the repetitive and time-consuming biological experiments and to develop software for the integrated management of the complex biological data. This is one of the revolutionary breakthroughs in the production of biological datasets. Notably, the ways of creating a desired genotype—built using DNA components through technology based on synthetic biology—and testing the resulting phenotype using automated robots have led to the unparalleled rapid production of labeled data. Amyris, an American company that specializes in synthetic biology, applied a method to produce new bacterial strains (with different genotypes) every three minutes and succeeded in commercializing 15 products through the course of seven years. Another synthetic biology startup company, Ginkgo Bioworks, attracted a total of 1 billion US dollars from investors for robot-assisted strain design technology in 2017. In addition, companies such as Zymergen and Counsyl are rapidly producing biological data via automated robots and refining the strains and proteins through deep learning, whereas companies like Transcriptic and Riffyn are developing a platform technology that will allow the rapid production and analysis of large quantities of highly complex biological data through the design of cloud-based synthetic biology software. While such private companies in the United States are rapidly developing innovative tools for biotechnology with plentiful capital and manpower, universities and research institutions in developed countries have been establishing Biofoundries with support from the respective Governments. For instance, a Biofoundry known as the Global Biofoundries Alliance, has been formed from 16 institutions from seven nations, for information sharing and the rapid development of automation-based synthetic biology technology [5].

The two fields, biology and statistics, naturally resemble each other. Most biological phenomena arise from the probability-based interactions among myriads of molecules, which can be explained through the statistical concepts of ‘probability’ and ‘error’. In other words, the procedure of getting a result through statistical inference is similar with that of phenotypic expression of a complex biological system in the point of allowing errors and stochasticity. Nonetheless, one of the reasons why AI models have not yet exerted a significant influence in the bioscience field is the lack of a sufficient scale of labeled data with low-speed data production. However, the developments of automated robots and software have enabled precise and rapid performances in the repetitive and time-consuming processes of biological experiments, implying the potential for large-scale labeled genomic data being established in the future. What seems clear is that, thanks to the development of sequencing technology and automated robots, the speed of biological data production has been reduced from ten or more years to within several days, and the future advancements in information technology will further enhance the speed. The improvement in AI models, in line with the accumulation of large-scale data, implies a preoccupancy of basic knowledge and intellectual property with respect to the field of life science with its complex and massive uncharted territories. The first artificial microorganism created by the J. Craig Venter Institute [6] and the artificial yeast synthesis project [7], which have excited the media into referring to them as the “realm of the God,” were the results of high-speed DNA synthesis technology based on automated robots. The relevant research groups have already acquired basic scientific knowledge of biological phenomena and set out toward research, where such knowledge is applied. The current AI algorithms and platform information technologies are mostly open-source based, which means anyone can easily apply the AI algorithms. Now, it might be possible to narrow the gap rapidly for the scientific technology of developed countries simply by collecting high-quality labeled data on a large-scale with a help of automated robots.
